# Targeting c-Jun in A549 Cancer Cells Exhibits Antiangiogenic Activity *In Vitro* and *In Vivo* Through Exosome/miRNA-494-3p/PTEN Signal Pathway

**DOI:** 10.3389/fonc.2021.663183

**Published:** 2021-04-09

**Authors:** Chen Shao, Yingying Huang, Bingjie Fu, Shunli Pan, Xiaoxia Zhao, Ning Zhang, Wei Wang, Zhe Zhang, Yuling Qiu, Ran Wang, Meihua Jin, Dexin Kong

**Affiliations:** ^1^ Tianjin Key Laboratory on Technologies Enabling Development of Clinical Therapeutics and Diagnostics, School of Pharmacy, Tianjin Medical University, Tianjin, China; ^2^ Department of Otorhinolaryngology Head and Neck, Institute of Otorhinolaryngology, Tianjin First Central Hospital, Tianjin, China; ^3^ School of Medicine, Tianjin Tianshi College, Tianyuan University, Tianjin, China

**Keywords:** c-Jun, exosomes, CRISPR/Cas9, lung cancer, miR-494, angiogenesis

## Abstract

The oncogene c-Jun is activated by Jun N-terminal kinase (JNK). Exosomes are nanometer-sized membrane vesicles released from a variety of cell types, and are essential for cell-to-cell communication. By using specific JNK inhibitor SP600125 or CRISPR/Cas9 to delete c-Jun, we found that exosomes from SP600125-treated A549 cancer cells (Exo-SP) or from c-Jun-KO-A549 cells (Exo-c-Jun-KO) dramatically inhibited tube formation of HUVECs. And the miR-494 levels in SP600125 treated or c-Jun-KO A549 cells, Exo-SP or Exo-c-Jun-KO, and HUVECs treated with Exo-SP or Exo-c-Jun-KO were significantly decreased. Meanwhile, Exo-SP and Exo-c-Jun-KO enhanced expression of phosphatase and tensin homolog deleted on chromosome ten (PTEN). Addition of miR-494 agomir in Exo-c-Jun-KO treated HUVECs inhibited PTEN expression and promoted tube formation, suggesting the target of miR-494 might be PTEN in HUVECs. Moreover, A549 tumor xenograft model and Matrigel plug assay demonstrated that Exo-c-Jun-KO attenuated tumor growth and angiogenesis through reducing miR-494. Taken together, inhibition of c-Jun in A549 cancer cells exhibited antiangiogenic activity *in vitro* and *in vivo* through exosome/miRNA-494-3p/PTEN signal pathway.

## Introduction

c-Jun, encoded by the c-*jun* proto-oncogene, appears to play a central role in cellular signal transduction and positively regulate cell proliferation through inhibiting tumor suppressor gene expression and function (1, 2). The Jun is positively autoregulated by its product Jun/AP-1 (2). AP-1 (activating protein-1) dimers include the Jun, Fos and activating transcription factor (ATF) subgroups of transcription factors that bind to a common DNA site (3). c-Jun activation requires an upstream molecule Jun N-terminal kinase (JNK), which binds to the c-Jun transactivation domain and phosphorylates at Ser63 and Ser73 (4).

Angiogenesis is the formation of new blood vessels from pre-existing ones. To support the growth or local metastasis of cancer cells, tumor tissue needs oxygen and nutrients provided by blood vessels (5). So suppression of tumor angiogenesis has become an essential strategy for cancer therapy.

Different cell types such as hematopoietic cells, primary and normal cells, and tumor cells release exosomes to epigenetically reprogram their neighboring cells (6). Exosomes are a class of extracellular vesicles defined as 40-100 nm diameter membrane, present with a characteristic cup-shaped morphology as observed by electron microscopy (7, 8). Exosomes contain proteins, nucleic acids, lipids and other bioactive molecules, which are shuttled from donor cells to the recipient cells (9). Tumor-derived exosomes could alter local and systemic microenvironment and therefore facilitate cancer cell proliferation, chemoresistance, and tumor angiogenesis (10).

Micro RNAs (miRNAs) are single stranded non-coding RNA with 19-25 nucleotides in length, which regulate gene expression at the post-transcriptional level with predominant mechanism of epigenetic regulation by binding sites in the 3’-untranslated region (UTR) of target messenger RNA (mRNA) (11). Numerous miRNAs are enriched in tumor-derived exosomes, and are transferred to endothelial cells to regulate angiogenesis (12).

However, the role of c-Jun in cancer cells in angiogenic effect of exosomes remained unreported. Recently, we found that Exosomes from SP600125 (JNK specific inhibitor)-treated non-small cell lung carcinoma (NSCLC) A549 cells (Exo-SP) inhibited human umbilical vein endothelial cells (HUVECs) tube formation. In the present study, we investigated the detailed mechanism of c-Jun in cancer cells to regulate angiogenesis through mediating exosome/miRNA/tensin homolog deleted on chromosome ten (PTEN) signal pathway.

## Methods

### Cell Culture

A549 cells were obtained from the Cell Resource Center, Peking Union Medical College (Beijing, China). A549 cells have been authenticated using STR profiling within the last three years and all experiments were performed with mycoplasma-free cells. A549 cells and c-Jun-KO A549 cells were cultured in RPMI 1640 medium containing 10% fetal bovine serum (FBS), 100 U/ml penicillin, and 100 μg/ml streptomycin. HUVECs were obtained from Lifeline Cell Technology (Frederick, MD, USA). Cell cultures were maintained in a humidified atmosphere with 5% CO_2_ at 37°C.

### Reagents

RPMI1640, DMEM, FBS, the enhanced chemiluminescence (ECL) reagent, and Total Exosome Isolation Reagents (from cells) were purchased from Thermo Fisher Scientific (Waltham, MA, USA). The antibodies specific for phosphatidylinositol 3- kinase (PI3K) 110β, phospho-Akt (Ser 473, #9271), phospho-Akt (Thr 308, #2965), Akt (#9272), β-actin (#4967) and the horseradish peroxidase-conjugated goat anti-rabbit secondary antibody (#7074) were purchased from Cell Signaling Technology, Inc. (Danvers, MA, USA). Ki-67 (27309-1-AP) was purchased from Proteintech (Rosemont, IL, USA). The antibody specific for c-Jun (sc-44) was obtained from Santa Cruz Biotechnology, Inc. (Dallas, TX, USA). The α-SMA specific antibody, SP600125, and PKH26 Red fluorescent cell linker kit were purchased from Sigma Chemicals (St. Louis, MO, USA). The antibody specific for phosphatase and PTEN (abs131550) was purchased from Absin Bioscience Inc. (Shanghai, China). The exosome specific primary antibodies including CD81, CD9 and CD63 were purchased from SBI System Biosciences (EXOAB-KIT-1, Palo Alto, CA, USA). The Matrigel was purchased from BD Biosciences (San Josè, CA, USA). Anti-CD31 (ab28364) antibody was purchased from Abcam (Cambridge, MA, USA).

### Cell Viability

Cell viability was assessed using MTT assay as we previously reported (13), with a small modification. Briefly, A549 cells were seeded onto a 96-well culture plate and incubated with various concentrations of SP600125 (10, 25, 50, 100 μM) for 24 h or 48 h. In the case of HUVECs, the cells were seeded onto a 96-well culture plate and incubated with various concentrations of Exo, Exo-SP600125, or Exo-c-Jun-KO for 24 h or 48 h, and then 20 μl of MTT (5 mg/ml) was added to each well. After 4 h of incubation, the formazan was dissolved in DMSO, and optical density (OD) at 490 nm was measured using microplate reader iMark (BIO-RAD, Hercules, CA, USA).

### Isolation of Exosomes

After cell cultures reached 80% confluent, A549 cells or c-Jun-KO A549 cells were washed with PBS and incubated with FBS free RPMI 1640 medium for 48 h, or were treated with SP600125 for 48 h in FBS free medium. Exosomes were isolated from the medium by Total Exosome Isolation Regent as described in the manufacture’s manual. Briefly, the harvested supernatants were filtered through 0.22 μm membrane to remove cells and debris, then concentrated using Amicon Ultra-15 100K centrifuge tube (MERCK Millipore). After transferring the cell-free culture media to a new tube, 0.5 volumes of the Total Exosome Isolation Reagent were added. After incubation at 4°C overnight, the suspension was centrifuged at 10,000×g for 1 h to remove the supernatant. The resulting exosomes were re-suspended in PBS and stored at 4°C to be available for use.

### Cell Migration Assay and Tube Formation Assay

The cell migration assay was performed as we reported previously (14), with a small modification. Confluent HUVEC monolayers were mechanically wounded with a pipette tip and washed with PBS to remove the debris. Then the monolayers were cultured in the RPMI 1640 medium containing 1% FBS. The wound healing was observed and the images were taken under inverted microscopy after 18 h.

The tube formation assay was carried out as reported by us (15), with a small modification. HUVECs were treated with Exo, Exo-SP, or Exo-c-Jun-KO for 24 h. Fifty microliters of growth factor-reduced Matrigel were added in the wells of 96-well plates and incubated at 37°C for 1 h. Then the pretreated HUVECs were re-suspended and added to the Matrigel. Images of tube formation were captured by inverted microscope after 3 h. For quantification, total tubular length and nodes of tubes per well were measured by computer-assisted image analysis using ImageJ software.

### Protein Extraction and Western Blot

Western blot analysis was carried out as we previously reported (16). Cells and exosomes were collected with lysis buffer, and the protein concentration of each sample was determined by the BCA protein assay kit. Equal amounts of proteins were run on sodium dodesyl sulfate-polyacrylamide gel electrophoresis (SDS-PAGE) and transferred to the PVDF membrane. After being blocked with 5% skim milk, the membranes were incubated with each primary antibody overnight at 4°C, and then incubated with the respective HRP-conjugated secondary antibody for 1 h at room temperature. The signals were detected with ChemiDoc™ XRS+ System (BIO-RAD, Hercules, CA, USA) after exposure to ECL reagent.

### Fluorescent Imaging of Exosome Uptake

The cellular uptake of exosomes was measured by fluorescence microscopy. Exosomes were labeled with PKH26 red fluorescent cell linker kit according to the manufacturer’s instructions, and then cultured with HUVEC for 3 h. After washing, HUVECs nuclei were stained with Hoechst for 15 min. Exosome uptake was measured using a fluorescence microscope.

### miRNA Isolation and Real-Time Quantitative Reverse Transcription-PCR (qRT-PCR) Assay

Total RNA from the cells and exosomes was isolated using the TRIzol reagent (Life Technologies, Carlsbad, CA, USA) or E.Z.N.A.™ miRNA Kit (Omega Bio-tek, Norcross, GA, USA) in accordance with the manufacturer’s instructions. First-strand cDNA was synthesized from RNA primed by oligo (dT) using M-MLV reverse transcriptase. The RT-qPCR assay for multiple genes was performed with the miScript SYBR Green PCR Kit by an CFX96™ Real-Time PCR Dectection System (BIO-RAD, Hercules, CA, USA). The primer of miRNA sequences is listed in **Table 1**. The expression levels of U6 snRNA were used to normalize the relative amount of miRNA (RT-Primer 5’-TTCACGAATTTGCGTGTCATC-3’, Forward 5’-CGCTTCGGCAGCACATATAC-3’, Reversed 5’-TTCACGAATTTGCGTGTCATC-3’). The fold-change of miRNA was calculated using the 2^−ΔΔCT^ method.

**Table 1:** List of miRNA primers.

miRNA	RT-Primer (5’-3’)	Forward (5’-3’)
hsa-miR-21	GTCGTATCCAGTGCAGGGTCCGAGGTATTCGCACTGGATACGACTCAACA	GCGCGTAGCTTATCAGACTGA
hsa-miR-23a	GTCGTATCCAGTGCAGGGTCCGAGGTATTCGCACTGGATACGACGGAAAT	GCGATCACATTGCCAGGG
hsa-miR-221	GTCGTATCCAGTGCAGGGTCCGAGGTATTCGCACTGGATACGACGAAACC	CGCGAGCTACATTGTCTGCTG
hsa-miR-222	GTCGTATCCAGTGCAGGGTCCGAGGTATTCGCACTGGATACGACACCCAG	GCGCGAGCTACATCTGGCTA
hsa-miR-449a	GTCGTATCCAGTGCAGGGTCCGAGGTATTCGCACTGGATACGACACCAGC	CGCGTGGCAGTGTATTGTTA
hsa-miR-494	GTCGTATCCAGTGCAGGGTCCGAGGTATTCGCACTGGATACGACGAGGTT	CGCGTGAAACATACACGGGA
hsa-miR-9	GTCGTATCCAGTGCAGGGTCCGAGGTATTCGCACTGGATACGACTCATAC	GCGCGTCTTTGGTTATCTAGCT
hsa-miR-34a	GTCGTATCCAGTGCAGGGTCCGAGGTATTCGCACTGGATACGACACAACC	CGCGTGGCAGTGTCTTAGCT
hsa-miR-125-3p	GTCGTATCCAGTGCAGGGTCCGAGGTATTCGCACTGGATACGACAGCTCC	GCGACGGGTTAGGCTCTTG
hsa-miR-125-5p	GTCGTATCCAGTGCAGGGTCCGAGGTATTCGCACTGGATACGACTCACAA	CGCGTCCCTGAGACCCTAAC
hsa-miR-126	GTCGTATCCAGTGCAGGGTCCGAGGTATTCGCACTGGATACGACCGCATT	CGCGTCGTACCGTGAGTAAT
hsa-miR-145	GTCGTATCCAGTGCAGGGTCCGAGGTATTCGCACTGGATACGACAGGGAT	CGGTCCAGTTTTCCCAGGA
hsa-miR-146a	GTCGTATCCAGTGCAGGGTCCGAGGTATTCGCACTGGATACGACAACCCA	CGCGTGAGAACTGAATTCCA
hsa-miR-148a	GTCGTATCCAGTGCAGGGTCCGAGGTATTCGCACTGGATACGACACAAAG	GCGCGTCAGTGCACTACAGAA
hsa-miR-152	GTCGTATCCAGTGCAGGGTCCGAGGTATTCGCACTGGATACGACCCAAGT	CGCGTCAGTGCATGACAGA
hsa-miR-497	GTCGTATCCAGTGCAGGGTCCGAGGTATTCGCACTGGATACGACACAAAC	GCGCAGCAGCACACTGTG
hsa-miR-519c	GTCGTATCCAGTGCAGGGTCCGAGGTATTCGCACTGGATACGACATCCTC	GCGCGAAAGTGCATCTTTTTA
hsa-miR-155	GTCGTATCCAGTGCAGGGTCCGAGGTATTCGCACTGGATACGACAACCCC	CGCGTTAATGCTAATCGTGATA
Reverse (5’-3’)	AGTGCAGGGTCCGAGGTATT	

### The Gene of c-Jun Knockout (KO) With CRISPR/Cas9 Gene Editing

We used the CRISPR/Cas9 to disrupt the c-Jun gene. The c-Jun is reported to contain one exon and to be located at chromosome 1 (The National Center for Biotechnology Information, NCBI). The gRNA sequences targets c-Jun on exon1, with target-sequence as TGCTCTGTTTCAGGATCTTG. A549 cells were transfected with pX330A-1x2 plasmid of the c-Jun-targeted gRNA encoding SpCas9 by Lipofectamine 3000. Following 72 h of puromycin selection, the cell culture was extended for another 96 h without puromycin, and the surviving clonal cells were sub-cultured into 96 well with a density of one cell/well. Afterward, cells were cultured for 7-10 days. Individual clones constructed with knockout of c-Jun were expanded and screened for c-Jun depletion by genomic DNA sequencing and Western blot.

### miRNA Agomir Transfection

HUVECs were transfected with miR-494 agomir (sense: 5’-UGAAACAUACACGGGAAACCUC-3’; anti-sense: 5’-GGUUUCCCGUGUAUGUUUCAUU-3’) using Lipofectamine 6000 transfection reagent for 24 h, according to the manufacturer’s instructions. All experimental control samples were treated with equal concentrations of a non-targeting control (NC) agomir (sense: 5’-UUCUCCGAACGUGUCACGUTT-3’; anti-sense: 5’-ACGUGACACGUUCGGAGAATT-3’). Chemically modified miR-494 agomir and NC agomir were purchased from Shanghai GenePharma Co., Ltd. Simultaneously, Exo or Exo-c-Jun-KO were added. After 24 h, cells were washed by PBS and collected to be available for use.

### Nude Mice Xenograft Tumor Experiments

BALB/c athymic nude mice (6 weeks old male) were kept in a specific pathogen-free environment. All animal experiments were conducted at Laboratory Animal Center of Institute of Radiation Medicine, the Chinese Academy of Medical Sciences in accordance with the Institutional Animal Care and Use Committee guidelines. Mice were injected subcutaneously (s.c.) in the flank with 1×10^7^ A549 cells. When subcutaneous tumors grew to 30 to 50 mm^3^, the mice were randomly divided into four groups, with six mice each. For exosome treatment, 2 μg of exosomes such as Exo, Exo-c-Jun-KO, and those treated with miR-494 agomir or agomir NC was injected intratumorally every other day for 14 days. Tumor volume was measured every other day. Mice were sacrificed by cervical dislocation 16 days after treatment, and tumor specimens were fixed with 4% formaldehyde, embedded in paraffin, and subjected to routine histological experiment. Immunohistochemistry (IHC) was carried out on 5-μm sections to visualize cells by using Ki-67, CD31, α-smooth muscle actin (α-SMA) antibodies. Six field images were collected using a Leica photomicroscope from three biopsies per specimen. Staining intensity was analyzed by Image-pro plus software.

### *In Vivo* Matrigel Plug Assay

HUVECs mixed with or without exosome, miR-494 agomir and Lipofectamine 6000, were added in 200 μl of High Concentration Matrigel™ Matrix and then subcutaneously injected into BALB/c nude mice (6 weeks old, male). After 14 days, mice were sacrificed and the Matrigel plugs were harvested for analysis. The degree of vascularization was evaluated by measuring hemoglobin content using the Hemoglobin test kit.

### Statistical Analysis

All data are expressed as means ± SD of triplicate values. One-way ANOVA followed by Tukey’s Multiple Comparison Test was utilized to determine the statistical significance with GraphPad Prism 5 (GraphPad, San Diego, CA, USA). A p value less than 0.05 was considered statistically significant.

## Results

### A549 Cells Showed High Sensitivity to JNK Inhibitor SP600125 With High Expression of c-Jun

**Figure 1:**
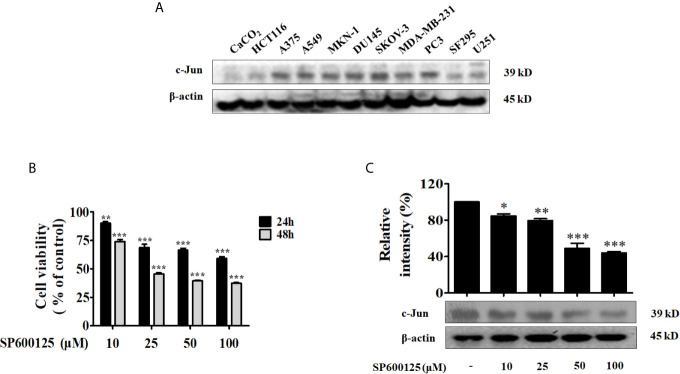
A549 was most sensitive to JNK inhibitor SP600125. **(A)** The expression of c-Jun in various tumor cells (CaCO_2_, HCT116, A375, A549, MKN-1, DU145, SKOV-3, MDA-MB-231, PC3, SF295, and U251) was determined by Western blot. **(B)** A549 cells were incubated with various concentrations (10, 25, 50, and 100 μM) of JNK inhibitor SP600125 for 24 h or 48 h. Cell viability was determined by MTT assay. **(C)** The cell lysates were collected and immunoblotted with antibody for c-Jun after treatment with 10, 25, 50, and 100 μM of SP600125 for 48 h, the relative ratios of c-Jun was calculated by analyzing immunoblot band intensities. Data show the mean ± SD of three independent experiments. *p < 0.05, **p < 0.01, and ***p < 0.001, compared to the non-treated HUVECs.

The transcription factor c-Jun is implicated with several cellular processes such as proliferation and cell transformation, and is up-regulated in numerous carcinomas. To identify the tumor cells with high level of c-Jun, we examined c-Jun protein expression in 11 human tumor cell lines including CaCO_2_ (colon cancer), HCT116 (colon cancer), A375 (melanoma cancer), A549 (non-small cell lung carcinama), MKN-1 (gastric cancer), DU145 (prostate cancer), SKOV-3 (ovarian cancer), MDA-MB-231 (breast cancer), PC3 (prostate cancer), SF295 (glioblastoma cancer), and U251 (glioblastoma cancer). Among them, A375, A549, MKN-1, DU145, SKOV-3, MDA-MB-231 and PC3 showed relatively higher expression of c-Jun (**Figure 1A**). Since the c-Jun activation domain is phosphorylated only by the JNKs, and the c-Jun promoter activity is autoregulated by c-Jun/AP-1, we treated the above 7 kinds of cancer cell lines with specific JNK inhibitor SP600125 to find out the most sensitive cancer cells to c-Jun. As a result, SP600125 showed most potent cytotoxic activity on A549 lung cancer cells (**Figure 1B** and **Figure S1**). SP600125 inhibits JNK activity and, thus, c-Jun phosphorylation (Ser-63, -73). In A549 cells, the expression of c-Jun was dose-dependently inhibited by SP600125 (**Figure 1C**).

### Exo-SP Inhibits Tube Formation and PTEN-Akt Pathway in the Recipient HUVECs

**Figure 2:**
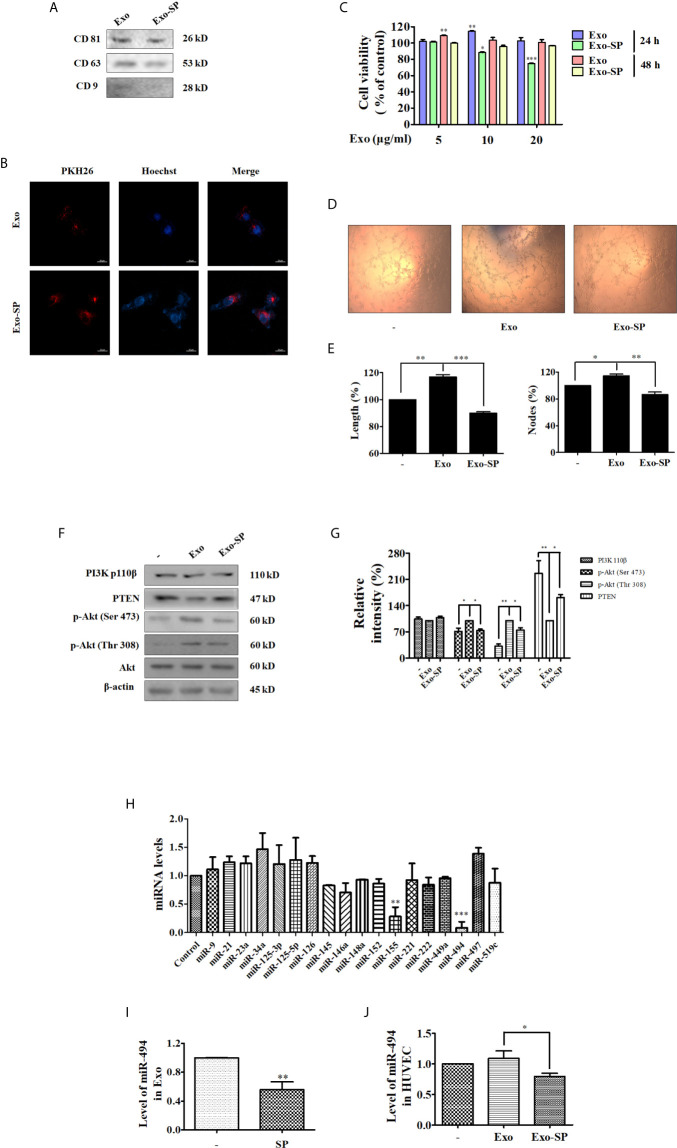
Exo-SP inhibits tube formation and suppresses PTEN/Akt pathway in the recipient HUVECs, and the levels of miRNAs in A549 or SP600125 treated A549 cells, Exo or Exo-SP, and HUVECs treated with Exo or Exo-SP **(A)** A549 cells were treated with or without 50 μM of SP600125 for 48 h in FBS free RPMI 1640 medium, and Exo and Exo-SP were isolated. Expression of exosomes markers CD81, CD63 and CD9 in the Exo and Exo-SP was detected by Western blot. **(B)** The isolated Exo and Exo-SP were labeled with PKH26 and Hoechst, and incubated with HUVECs for 3 h. The cellular uptake of Exo or Exo-SP was observed under fluorescent microscope. **(C)** HUVECs were treated with different concentrations (5, 10, 20 μg/ml) of Exo or Exo-SP for 24 h or 48 h, respectively. Cell viability was determined by MTT assay. **(D)** HUVECs were treated with Exo or Exo-SP for 24 h, and then seeded on Matrigel. Formation of tube-like structures was examined 3 h later. **(E)** Quantitative data for the tube-like structures were obtained by analyzing Length (%) and Nodes (%) density with Image J software. **(F)** HUVECs were treated with Exo or Exo-SP for 24 h, and then collected and lysed for immunoblot with specific antibodies. **(G)** Quantification of the results in **(F)**. **(H)** The levels of various miRNAs in A549 cells treated with or without SP600125 for 48 h. **(I)** The level of miR-494 in Exo and Exo-SP. Exosomes isolated from A549 cells were treated with or without SP600125 for 48 h, to be available for miR-494 analysis. **(J)** The level of miR-494 in HUVECs treated with Exo or Exo-SP for 24 h. Data show the mean ± SD of three independent experiments. *p < 0.05, **p < 0.01, and ***p < 0.001.

Tumor-derived exosomes induce alterations in their recipient cells, thereby play crucial roles in tumor growth, metastasis and angiogenesis (17, 18). Firstly, A549 cells were incubated in FBS-free RPMI 1640 medium for 24 h, and treated with or without 50 μM of SP600125 for 48 h. Then we isolated exosomes from A549 cells and prepared Exo and Exo-SP lysates for immunoblot with antibodies of CD81, CD63 and CD9, which are known as exosome markers (**Figure 2A**). We further examined whether Exo and Exo-SP from tumor cells affect endothelial cells, by performing endothelial cell exosome uptake, proliferation assay, and tube formation assay. The confocal imaging in **Figure 2B** suggested that Exo and Exo-SP were successfully uptaken by HUVECs.

Angiogenesis process involves the proliferation, migration, and tube formation of endothelial cells, allowing subsequent new vessel growth toward tumor (19). To investigate the influence of Exo or Exo-SP on proliferation of endothelial cells, HUVECs were treated with Exo or Exo-SP at concentration of 5, 10 and 20 μg/ml for 24 h or 48 h. Then the percentage of viable cells was measured using MTT assay. As a result, the percentage of viable cells was elevated by Exo, and reduced by Exo-SP (**Figure 2C**). The treatment of Exo-SP for 24 h was more effective than that for 48 h, and the effect of 5 μg/ml was too weak while that of 20 μg/ml too strong. Therefore, in order to eliminate the influence of exosome-induced cell cytotoxicity, we used the 10 μg/ml of exosome to evaluate the effect on HUVECs in the following study.

In tube formation assay, HUVECs were pre-incubated with Exo or Exo-SP for 24 h, and then seeded into matrigel. Three hours later, tube formation was photographed. Treatment with Exo promoted the tube formation, which was reduced by Exo-SP (**Figures 2D, E** **)**.

In order to investigate angiogenetic or anti-angiogenetic effects of Exo or Exo-SP, we detected the angiogenesis-related proteins such as PI3K/Akt pathway proteins by Western blot, since PI3K/Akt pathway could activate endothelial cell proliferation and migration (20). After treatment for 24 h, Exo decreased the level of PTEN and increased the phosphorylation of Akt, without effect on PI3K 110β. In contrast, Exo-SP increased the level of PTEN and decreased the phosphorylation of Akt (**Figures 2F, G** **)**.

### The Level of miR-494 Was Reduced in SP-Treated A549 Cells, Exo-SP and Exo-SP-Treated HUVECs

Exosomes isolated from human islets were found to carry a subset of miRNAs. The miRNAs have become attractive because they were reported to post-transcriptionally down-regulate target mRNAs that initiate or facilitate the development of several diseases. Particularly, miRNAs play crucial roles in vascular remodeling (21). In this study we examined the levels of 18 human miRNAs such as miR-9, miR-21, miR-23a, miR-34a, miR-125-3p, miR-125-5p, miR-126, miR-145, miR-146a, miR-148a, miR-152, miR-155, miR-221, miR-222, miR-449a, miR-494, miR-497, miR-519c, which were reported to target genes that are involved in vascular remodeling processes (22–30). In order to determine whether treatment of SP600125 leads to miRNA variation, we evaluated the levels of miRNAs in SP600125 treated A549 cells, Exo-SP, and Exo-SP treated HUVECs. A549 cells were treated with or without SP600125 for 48 h, and then the miRNAs were isolated. qRT-PCR results showed that the expressions of miR-494 and miR-155 were significantly lower in SP600125 treated A549 cells relative to non-treated group, and there were no obvious changes of other miRNAs (**Figure 2H**). Between them, miR-494 was the more significantly decreased, therefore, we investigated the effect of miR-494 in the following study. A549 cells were treated with or without SP600125 for 48 h, and then miRNAs were isolated from Exo or Exo-SP. As shown in **Figure 2I**, the level of miRNA-494 was significantly reduced in Exo-SP compared with Exo. Furthermore, miR-494 level was also decreased in HUVECs treated with Exo-SP compared to the Exo treated group (**Figure 2J**).

### Exo-c-Jun-KO Inhibits Angiogenesis Through Down-Regulation of Angiogenesis Related Signal Proteins in HUVEC Cells

**Figure 3:**
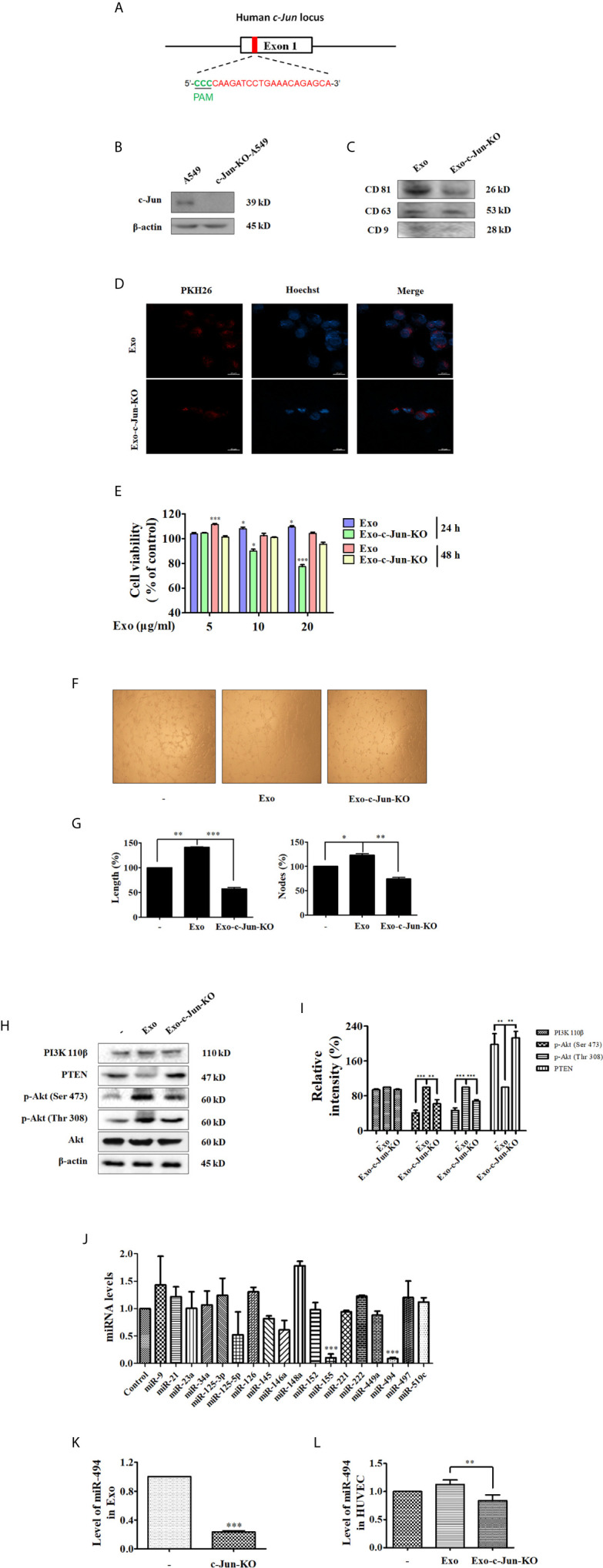
Exo-c-Jun-KO inhibits angiogenesis in HUVECs, and the levels of miRNAs in A549 and c-Jun-KO A549 cells, Exo and Exo-c-Jun-KO, and HUVECs treated with Exo and Exo-c-Jun-KO **(A)** Diagram of target site of c-Jun. gRNA targeting site is at exon 1 (red), protospacer adjacent motif (PAM) sequence (green). **(B)** Gene edited c-Jun-KO-A549 cells does not express the c-Jun. **(C)** The expression of CD81, CD63 and CD9 in Exo and Exo-c-Jun-KO. **(D)** Exosomes uptake determined by fluorescence staining assay. Exo and Exo-c-Jun-KO were labeled with PKH26, and then cultured with HUVECs for 3 h. HUVEC nuclei were labeled with Hoechst. **(E)** Cell viability of HUVECs after treated with Exo or Exo-c-Jun-KO for 24 h or 48 h. **(F)** Tube formation of HUVECs after treated with Exo or Exo-c-Jun-KO for 24 h. **(G)** Length (%) and Nodes (%) of tube formation were determined by Image J software. **(H)** The expression of PI3K p110β, PTEN, and phosphorylation of Akt in Exo or Exo-c-Jun-KO treated HUVECs. **(I)** Quantification of the results in **(H)**. **(J)** The levels of various miRNAs in A549 or c-Jun-KO A549 cells. **(K)** The level of miR-494 in Exo and Exo-c-Jun-KO. Exosomes isolated from A549 or c-Jun-KO A549 cells were cultured with FBS free RPMI 1640 medium for 48 h, to be available for miR-494 analysis. **(L)** The level of miR-494 in HUVECs treated with Exo or Exo-c-Jun-KO for 24 h. Data show the mean ± SD of three independent experiments. *p < 0.05, **p < 0.01, and ***p < 0.001.

In order to verify whether c-Jun affects the expression of miR-494 in A549 cell derived exosomes, thereby the angiogenesis of HUVECs, we used CRISPR/Cas9 to knockout the c-Jun in A549 cells (c-Jun-KO A549). Diagram of c-Jun gene exon 1 of gRNA targeting site is shown in **Figure 3A**. The sequence character of exon 1 in c-Jun was confirmed in A549 cells with Sanger sequencing carrying a 1-bp addition at the gRNA-targeting region (**Figure S2**). In addition, Western blot further confirmed no expression of c-Jun in gene-edited A549 cells (**Figure 3B**).

The isolated particles from c-Jun-KO A549 cells were confirmed to be exosomes, by examining the expression of the exosome markers including CD81, CD63 and CD9 (**Figure 3C**). Confocal images in **Figure 3D** showed that Exo-c-Jun-KO was uptaken by HUVECs. To confirm whether Exo-c-Jun-KO could exhibit anti-angiogenic effect on HUVECs as Exo-SP, we used MTT assay to determine the cell viability of HUVECs firstly. As shown in **Figure 3E**, the results were consistent with results of Exo-SP. Exo promoted, but Exo-c-Jun-KO suppressed, HUVEC proliferation, relative to untreated control for 24 h. However, treatment with Exo-c-Jun-KO for 48 h did not alter cell viability obviously. Furthermore, we investigated the effect of Exo-c-Jun-KO on tube formation in HUVECs. After incubation for 24 h, the length and nodes of tube were reduced by Exo-c-Jun-KO compared with the Exo-treated group (**Figures 3F, G** **)**. Meanwhile, Exo-c-Jun-KO decreased the Exo elevated phosphorylation of Akt, but enhanced the expression of PTEN, without obvious change of PI3K 110β (**Figures 3H, I** **)**. These results are consistent with Exo-SP treatment, indicating that Exo-c-Jun-KO might suppress angiogenesis through PTEN-Akt pathway.

### The Level of miR-494 Was Reduced in c-Jun-KO-A549 Cells, Exo-c-Jun-KO and HUVECs Treated With Exo-c-Jun-KO

Exosomal miRNAs could stimulate angiogenesis and facilitate metastasis in cancer (31). To verify whether c-Jun acts on the exosomal miRNAs to influence endothelial angiogenesis, we measured the miRNA levels in c-Jun-KO-A549 cells, Exo-c-Jun-KO, and Exo-c-Jun-KO treated HUVECs. The qRT-PCR analysis showed only miR-494 and miR-155 changed significantly in c-Jun knockout A549 cells (**Figure 3J**). Consistent with Exo-SP, miR-494 was more reduced than miR-155. In addition, we also found c-Jun-KO-related reduction of miR-494 level in Exo-c-Jun-KO and Exo-c-Jun-KO treated HUVECs, respectively (**Figures 3K, L** **)**.

### Exosomal miR-494 Derived From c-Jun-KO-A549 Cells Inhibits Angiogenesis Through Targeting PTEN in Recipient HUVECs

**Figure 4:**
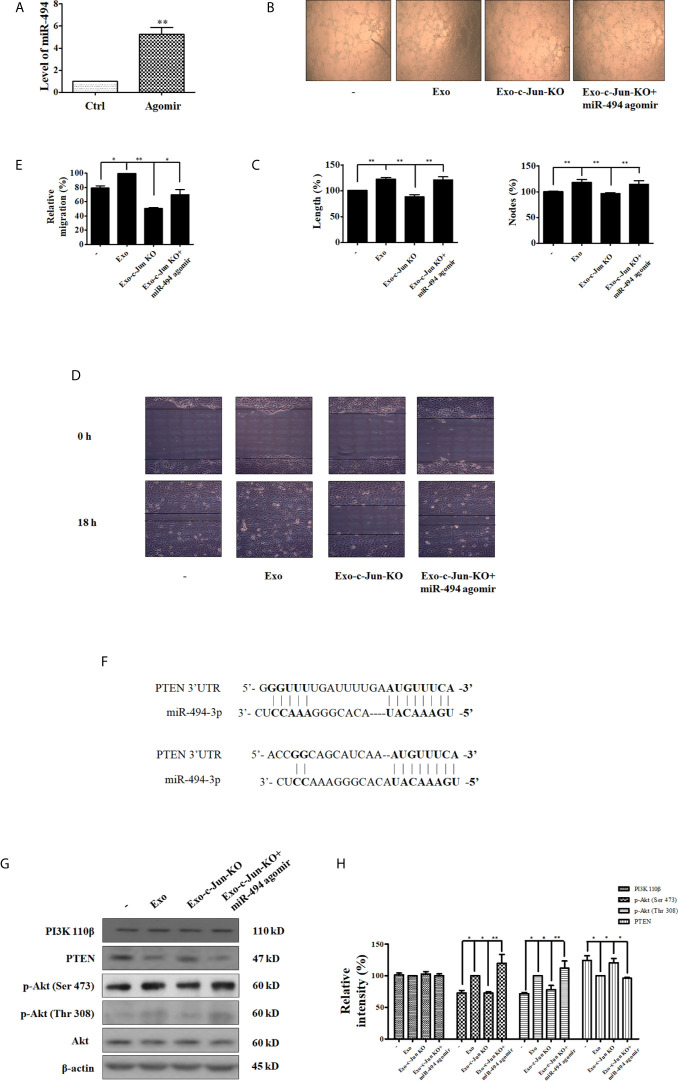
miR-494/PTEN is involved in angiogenesis inhibited by Exo-c-Jun-KO **(A)** The level of miR-494 in HUVECs transfected with the miR-494 agomir or agomir NC. **(B)** Tube formation of HUVECs treated with Exo, Exo-c-Jun-KO, and those simultaneously transfected with miR-494 agomir or agomir NC. **(C)** Length (%) and Nodes (%) were determined by Image J software. **(D)** Migration of HUVECs treated with Exo, Exo-c-Jun-KO, and those simultaneously transfected with miR-494 agomir or agomir NC. **(E)** Quantification of the results in **(D)**. **(F)** PTEN as the target gene of miR-494. **(G)** Changes of PTEN/Akt related proteins in HUVECs treated with or without Exo or Exo-c-Jun-KO, and those simultaneously transfected with miR-494 agomir or agomir NC. **(H)** Quantification of the results in **(G)**. Data show the mean ± SD of three independent experiments. *p < 0.05 and **p < 0.01.

To investigate the effect of miR-494 on tumor-angiogenesis, we established HUVECs with over expression of miR-494 by miR-494 agomir transfection, and then performed the tube formation assay and wound healing experiments. Firstly, HUVECs were transfected with miR-494 agomir or their scramble control agomir NC. qRT-PCR revealed that the transfection increased level of miR-494 to be 5 times over the agomir NC group (**Figure 4A**), indicating that the miR-494 agomir was successfully transfected in HUVECs. Tube formation results showed that Exo-c-Jun-KO plus miR-494 agomir in HUVECs significantly elevated tubular length and nodes compared with the cells treated with Exo-c-Jun-KO plus agomir NC (**Figures 4B, C** **)**. In addition, Exo-c-Jun-KO inhibited endothelial cell migration compared with the Exo treated group, and Exo-c-Jun-KO plus miR-494 agomir increased endothelial cell migration compared with the Exo-c-Jun-KO treated group (**Figures 4D, E** **)**.

The binding site between miR-494 sequence and PTEN sequence is shown in **Figure 4F**, indicating that PTEN coding sequence might be a potential target of miR-494. And several previous reports have shown that miR-494 directly targets PTEN (32–34). To demonstrate the target protein of miR-494 from Exo-c-Jun-KO, we investigated the effect of miR-494 on Akt pathway. HUVECs were cultured with Exo, Exo-c-Jun-KO, or Exo-c-Jun-KO plus miR-494 agomir for 24 h, and the expression and phosphorylation of the PI3K/Akt pathway proteins were detected using Western blot. PTEN is the negative regulator of the PI3K/Akt oncogenic signaling pathway, thereby inhibiting uncontrolled cell survival, growth and migration (35). PTEN has been reported as a target gene of miR-494, therefore, we verified that PTEN was down-regulated by miR-494 agomir in HUVECs. Exo-c-Jun-KO plus miR-494 agomir led to lower levels of PTEN expression compared with Exo-c-Jun-KO group. However, the phosphorylation of Akt was increased in Exo-c-Jun-KO plus miR-494 agomir treated group compared with Exo-c-Jun-KO (**Figures 4G, H** **)**. These findings demonstrated that the PTEN was the target gene of miR-494 in HUVECs.

### Exo-c-Jun-KO Suppressed A549 Lung Cancer Growth and Angiogenesis *In Vivo*

**Figure 5:**
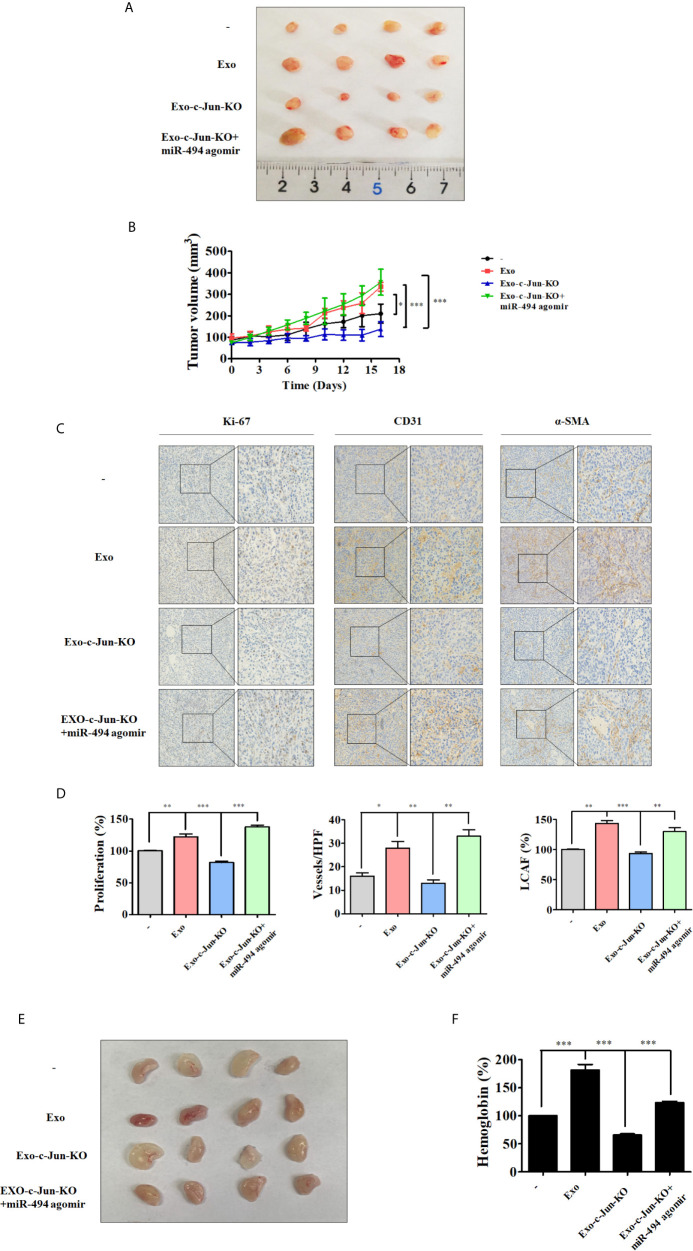
Exo-c-Jun-KO suppresses lung cancer growth and angiogenesis *in vivo* A549 cells were inoculated subcutaneously in the flank site of nude mice. Exo, Exo-c-Jun-KO, miR-494 agomir were intratumorally injected every other day for 14 days. After 16 days, the tumors were excised from mice. **(A)** Representative images of the tumors removed from nude mice. **(B)** Quantification of tumor volume. Tumor volumes were measured every other day starting from the day before the first treatment. **(C, D)** Immunohistochemical staining of Ki-67, CD31 and α-SMA in tumor tissues of different groups and their quantification. **(E)** Photos of the matrigel plugs from nude mice after various treatments. **(F)** Hemoglobin level in the matrigel plugs. Data show the mean ± SD (n=4). *p < 0.05, **p < 0.01, and ***p < 0.001.

We used mouse xenograft model to investigate whether miR-494 from Exo-c-Jun-KO could affect tumor growth *in vivo*. A549 cells were subcutaneously inoculated into the flanks of nude mice. And Exo, Exo-c-Jun-KO, or Exo-c-Jun-KO plus miR-494 agomir was intratumoral administered every other day for 14 days. Mice were sacrificed and the representative tumor images were shown in **Figure 5A**. The tumors from the Exo group were significantly larger than non-treated control group, and those from the Exo-c-Jun-KO group were smaller than Exo group. Tumors from Exo-c-Jun-KO plus miR-494 group were larger than Exo-c-Jun-KO group. The tumor growth pattern showed similar results (**Figure 5B**). Accordingly, the immunohistochemical analysis showed that Exo treated tumors had a higher number of cells positive for the proliferative marker Ki-67 than control group, and Exo-c-Jun-KO had shown less number of cancer cells that Exo group, and Exo-c-Jun-KO plus miR-494 had higher number of cells than Exo-c-Jun-KO group (**Figures 5C, D** **)**. In addition, tumors from Exo group also had more cells positive for endothelial cell marker CD31, with stronger staining than control group, and Exo-c-Jun-KO treatment had lower level of CD31 in the xenograft tumor than Exo treatment. Fibroblasts are important component of the tumor stroma, which are thought to be critical driver of tumor progression in a number of organs (36). We found that the higher levels of α-SMA, a cancer (carcinoma)-associated fibroblasts (CAF) marker, positive stromal cells were detected in Exo treatment group, and Exo-c-Jun-KO treatment showed lower levels of α-SMA (**Figures 5C, D** **)**.

### Exo-c-Jun-KO Suppressed A549 Lung Cancer Angiogenesis *In Vivo*

To further investigate the anti-angiogenic effect of Exo-c-Jun-KO, we examined the effect of Exo-c-Jun-KO by use of Matrigel plug assay *in vivo*. HUVECs mixed with exosomes, agomir and Matrigel were subcutaneously injected into nude mice, and the vasculature was examined. Consistent with the *in vitro* and above *in vivo* results, the photo of Matrigel plugs showed that Exo-stimulated vascularization was markedly suppressed by Exo-c-Jun-KO (**Figure 5E**). ELISA result showed that Exo accumulated hemoglobin, while Exo-c-Jun-KO treatment led to decrease of hemoglobin content (**Figure 5F**). And treatment of Exo-c-Jun-KO plus miR-494 agomir reversed the reduction by Exo-c-Jun-KO.

## Discussion

c-Jun was reported as an oncoprotein, of which the overexpression greatly enhanced the tumorigenic properties of the MCF7 human breast cancer cell line (37). c-Jun is highly expressed in tumor cells of patients with classical Hodgkin’s disease (38), and the number and size of hepatic tumors were significantly attenuated when c-*jun* was inactivated (39). In the present study, we found that tumor cell lines including A375, A549, MKN-1, DU145, SKOV-3, MDA-MB-231 and PC3 expressed the c-Jun protein. JNK phosphorylates and regulates the activity and expression of the Jun proteins (2, 4). Therefore, we used JNK specific inhibitor SP600125 to find out JNK sensitive cell line. Among the above 7 tumor cell lines, NSCLC A549 was most sensitive, and therefore was selected to investigate the c-Jun related mechanism.

Exosomes are a class of extracellular vesicles released by various cell types, act as a mediator of cell-to-cell communication (31). In this study, exosomes derived from A549, SP600125 treated A549, or c-Jun-KO-A549 cells were successfully isolated and characterized. Tumor-derived exosomes reprogram endothelial cells by ligand/receptor signaling, miRNA and RNA transfer after fusion with the plasma membrane, internalization through phagocytosis, endocytosis, micropinocytosis or lipid raft-mediated internalization (12). In this study we found that PKH26-labeled Exo, Exo-SP, Exo-c-Jun-KO were localized in the cytoplasm of HUVECs, implying that exosomes could be internalized by HUVECs.

Most cancer cells release exosomes, which dictate the behavior of the recipient cells for the ultimate benefit of the cancer cells (18, 40). Blood vessels deliver oxygen and essential nutrients to nourish cancers. Neovascularization promotes tumor extension and invasion into nearby normal tissue, as well as distant metastasis to form new colonies of cancer cells (5, 19). Therefore, angiogenesis plays an important role in tumor growth and metastasis. It was reported that exosomes from Chronic Myelogenous Leukemia (CML) cells modulate the process of neovascularization by directly affecting endothelial cells (41); exosomes from glioblastoma multiforme (GBM) cells growing in hypoxic induce angiogenesis by phenotypic modulation of endothelial cells (42). However, the role of c-Jun in exosome-mediated angiogenesis has not been reported. In the present study, we found that angiogenesis of HUVECs was enhanced after stimulated by Exo. However, Exo-SP and Exo-c-Jun-KO inhibited HUVEC tube formation and proliferation. These results suggest that down-regulaton of c-Jun in tumor cells reduced the vascular development caused by Exo in the neighborhood of the tumor.

Cancer exosomes carry malignant information in the form of miRNA, mRNA, DNA fragment and proteins that can reprogram recipient cells (43). miRNAs are small, endogenous, conserved, single-stranded, non-coding RNAs, which degrade target mRNAs or inhibit translation post-transcriptionally (21). Recently, exosomal miRNAs and their relation with cancer have been frequently reported. Among which, exosomal miR-135b from hypoxic multiple myeloma cells enhances angiogenesis by targeting factor-inhibiting hypoxia-inducible factor 1 (44); miR-23a from nasopharyngeal carcinoma derived exosomes mediated angiogenesis by targeting testis-specific gene antigen (45). In this regard, we investigated variation of miRNA levels in A549 cells, exosomes and exosomes treated HUVECs, and found that level of miR-494 was reduced in A549 cells with SP treatment or c-Jun-KO compared with non-treated group. And the level of miR-494 was down-regulated in Exo-SP and Exo-c-Jun-KO. In addition, incubation with Exo-SP and Exo-c-Jun-KO showed lower level of miR-494 compared with Exo in recipient HUVECs. These results revealed that miR-494 level might be involved in the Exo-SP and Exo-c-Jun-KO mediated angiogenesis behaviors of HUVECs.

Angiogenesis is a multistep process controlled by the balance of pro- and anti-angiogenesis factors. PI3K/Akt pathway is considered to play an important role in tumorigenesis (46). PI3K regulates cell-cycle progression, protein synthesis, cell growth, and angiogenesis mainly through Akt (46). In the present study we found that Exo-SP and Exo-c-Jun-KO decreased the phosphorylation of Akt elevated by Exo. However, the level of PI3K 110 was unchanged. The second messenger phosphatidylinositol 3,4,5-triphosphate (PIP3) is produced through phosphorylation of phosphatidylinositol 4,5-bisphosphate (PIP2) catalyzed by PI3K. The PTEN dephosphorylates PIP3 to PIP2, acting thereby as a direct antagonist of PI3K (47). PTEN is known as a tumor suppressor and appears to be mutated at considerable frequency in human cancers (48). PTEN has been reported to be lowly expressed in lung cancer (49). In this study we found that the anti-angiogenesis efficacy of Exo-SP and Exo-c-Jun-KO might be mediated *via* elevation of PTEN expression in HUVECs. We also found that increase of miR-494 by the miR-494 agomir resulted in the reversal of the anti-angiogenesis effect of Exo-c-Jun-KO including tube formation and migration. Exo-c-Jun-KO plus miR-494 agomir further flipped the effect of Exo-c-Jun-KO on phosphorylation of Akt. miR-494 activates the PI3K/Akt pathway by targeting of PTEN and hence not only enhances the ability of myeloid-derived suppressor cells (MDSCs) to infiltrate into tumor tissue but also facilitates tumor invasion and metastasis (50). Low PTEN expression and high miR-494 expression are associated with high proliferation, low differentiation of tumor tissues and high possibility of early invasion and metastasis in NSCLC (51). In this study we verified that PTEN was downregulated by miR-494 agomir compared with the non-treated control group (Data not shown), and the expression of PTEN was decreased in HUVECs by Exo-c-Jun-KO plus miR-494 agomir compared with Exo-c-Jun-KO treated group. These results suggest that the reduced exosomal miR-494 from c-Jun-KO-A549 cells regulate the angiogenesis though up-regulation of expression of PTEN, which inactivated Akt and the downstream pathway in HUVECs.

Exo-c-Jun-KO blocked tumor growth and reduced tumor volume by approximately 57.8% compared with Exo treated mice, and Exo-c-Jun-KO plus miR-494 agomir converted the tumor growth. CD31 was used as a marker to indicate vascularization or blood vessel formation within tumor. CAFs, recognized by expression of α-SMA, are known to promote malignant growth, angiogenesis, invasion and metastasis (52). Immunohistochemical analysis showed that Exo-c-Jun-KO suppressed Ki-67, CD31 and α-SMA expression in the tumor section, and Exo-c-Jun-KO plus miR-494 agomir could neutralize such effect. These data indicated that low miR-494 expression in Exo-c-Jun-KO is correlated with lung cancer progression. Matrigel plug is a classic model to assess angiogenesis *in vivo*. In this work, Exo-c-Jun-KO decreased blood vessel formation in the Matrigel plug model compared with the Exo treated mice. And the blood vessels were increased after addition of miR-494 agomir. These results further revealed that Exo-c-Jun-KO possessed the vascular recruitment and organization through miR-494.

## Conclusions

**Figure 6:**
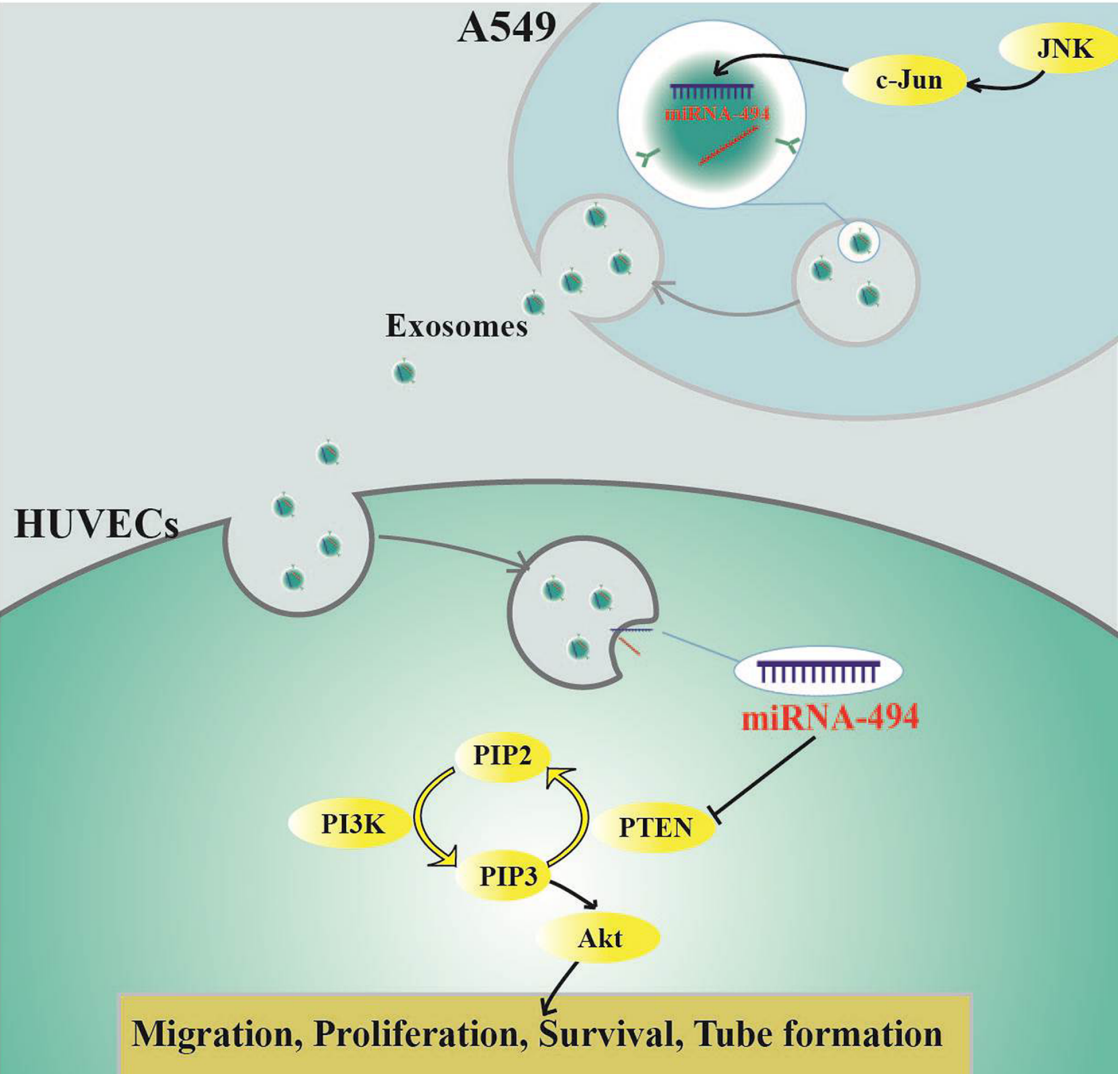
Schematic diagram indicating how c-Jun affects angiogenesis in HUVECs through exosome/miR-494/PTEN signal pathway.

In conclusion, we first elucidated that miR-494 was reduced in Exo-c-Jun-KO and Exo-SP, resulting in elevation of PTEN expression and down-regulation of Akt pathway in Exo-c-Jun-KO and Exo-SP treated HUVECs, which might contribute to the suppression of tumor angiogenesis. Our data demonstrated that Exo-c-Jun-KO attenuates the lung cancer angiogenesis by transferring exosomal miR-494 to recipient endothelial cells (**Figure 6**), suggesting targeting oncogene c-Jun might be a promising therapeutic approach for lung cancer.

## Data Availability Statement

The original contributions presented in the study are included in the article/**Supplementary Material**. Further inquiries can be directed to the corresponding authors.

## Ethics Statement

The animal study was reviewed and approved by Laboratory Animal Center of Institute of Radiation Medicine, the Chinese Academy of Medical Sciences.

## Author Contributions

MJ and DK designed the experiments and acquired funding for the study. CS, YH, BF, SP, XZ, and NZ performed the experiments. WW, ZZ, RW, and YQ provided technical assistances. CS and MJ wrote the manuscript. DK edited the manuscript. All authors contributed to the article and approved the submitted version.

## Funding

This study was supported by grants from the National Natural Science Foundation of China (81672809, 81673464, 81373441), grant for Major Project of Tianjin for New Drug Development (17ZXXYSY00050).

## Conflict of Interest

The authors declare that the research was conducted in the absence of any commercial or financial relationships that could be construed as a potential conflict of interest.
