# Supplementary figures and images for: Targeting c-Jun in A549 Cancer Cells Exhibits Antiangiogenic Activity In Vitro and In Vivo Through Exosome/miRNA-494-3p/PTEN Signal Pathway
Source: Front Oncol. 2021 Apr 9;11:663183. doi: 10.3389/fonc.2021.663183 (PMC8062808; doi:10.3389/fonc.2021.663183)

## Slide 1
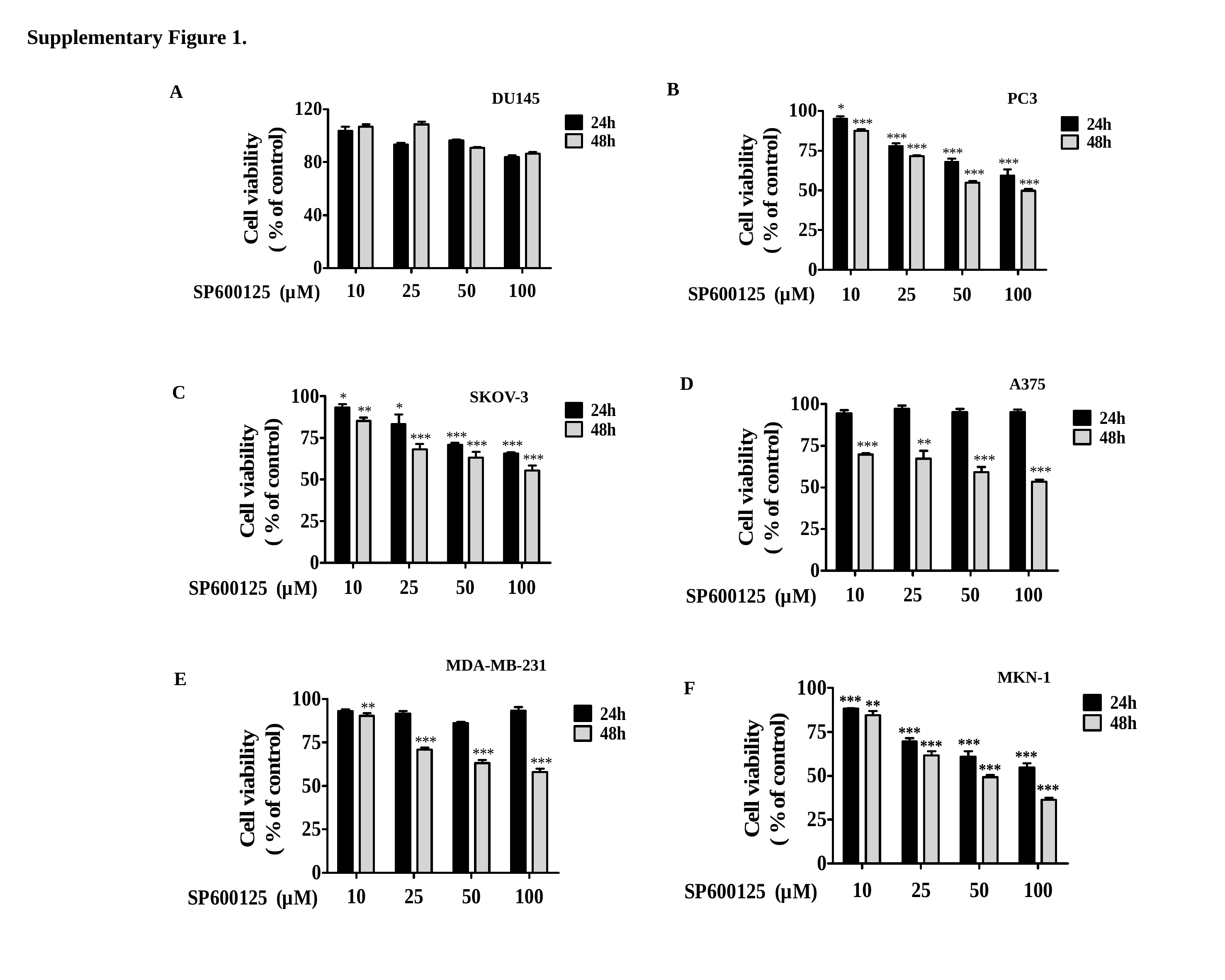

Supplementary Figure 1.
B
A
DU145
PC3
D
A375
C
SKOV-3
MDA-MB-231
E
MKN-1
F

## Slide 2
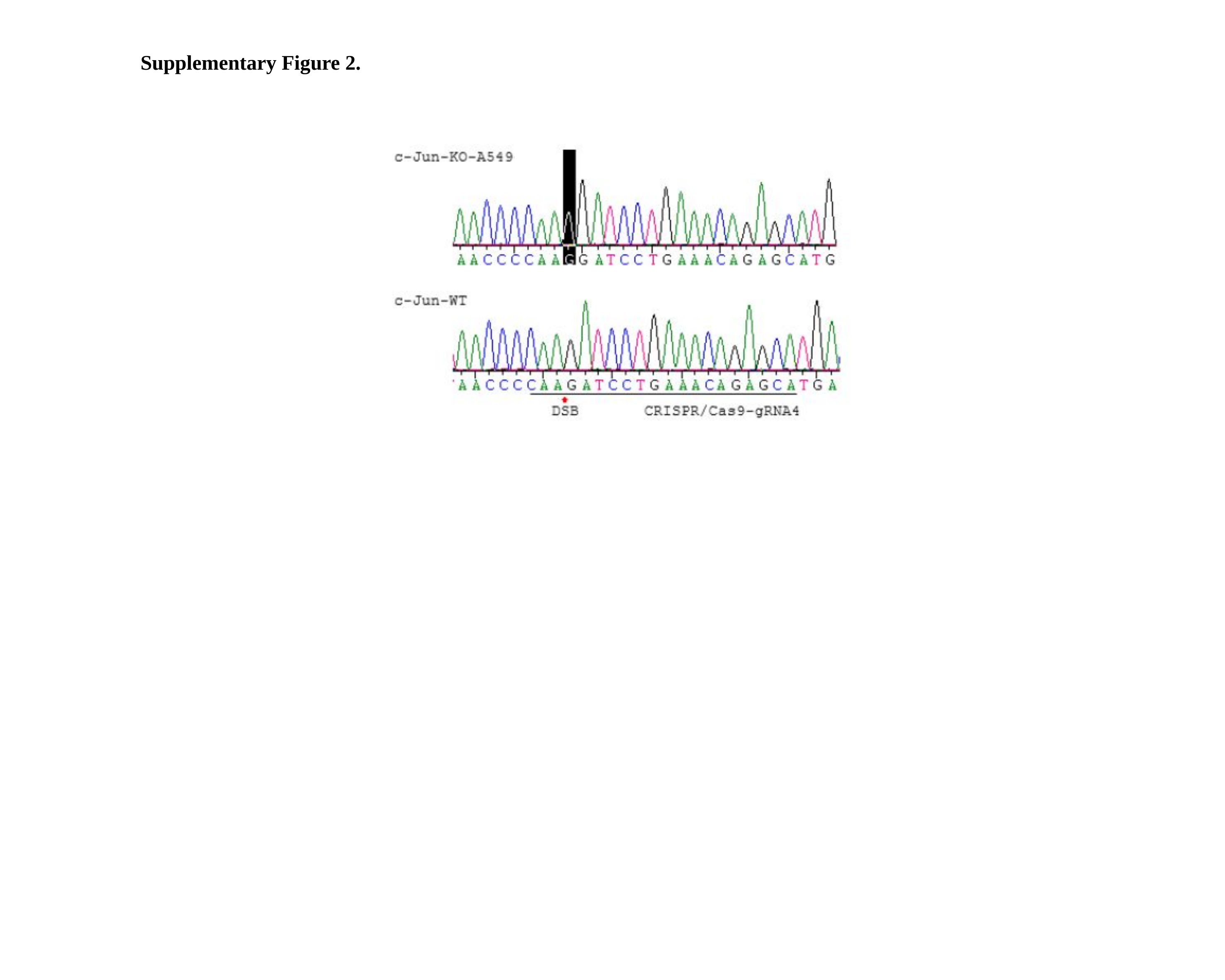

Supplementary Figure 2.

Supplement: Supplementary file 1 [file Presentation_1.pptx]
