# Supplementary material for: Targeting c-Jun in A549 Cancer Cells Exhibits Antiangiogenic Activity In Vitro and In Vivo Through Exosome/miRNA-494-3p/PTEN Signal Pathway
Source: Front Oncol. 2021 Apr 9;11:663183. doi: 10.3389/fonc.2021.663183 (PMC8062808; doi:10.3389/fonc.2021.663183)
Supplement: Supplementary file 2 [file Table_1.docx]

**Targeting c-Jun in A549 cancer cells exhibits antiangiogenic activity in vitro and in vivo through exosome/miRNA-494-3p/PTEN signal pathway**

Chen Shao^1^, Yingying Huang^1^, Bingjie Fu^1^, Shunli Pan^1^, Xiaoxia Zhao^1^, Ning Zhang^1^, Wei Wang^2^, Zhe Zhang^1^, Yuling Qiu^1^, Ran Wang^1^, Meihua Jin^1,*^, Dexin Kong^1,3,*^

^1^Tianjin Key Laboratory on Technologies Enabling Development of Clinical Therapeutics and Diagnostics, School of Pharmacy, Tianjin Medical University, Tianjin 300070, China;

^2^Department of Otorhinolaryngology Head and Neck, Institute of Otorhinolaryngology, Tianjin First Central Hospital, Tianjin 300192, China

^3^School of Medicine, Tianjin Tianshi College, Tianyuan University, Tianjin 301700, China.

*Correspondence: jinmeihua@tmu.edu.cn (M. Jin), kongdexin@tmu.edu.cn (D. kong)

**Figure legends**

**Figure S1.** The inhibitory activity of JNK inhibitor SP600125 on cell viability of several tumor cell lines. DU145, PC3, SKOV-3, A375, MDA-MB-231, MKN-1 cells were treated with various concentrations (10, 25, 50, and 100 μM) of SP600125 for 24 h and 48 h. Cell viability was determined by MTT assay.

**Figure S2.** The Sanger sequencing of PCR product in c-Jun-KO-A549 cells. The top sequence indicates occurrence of the 1-bp insertion at the gRNA-targeting region; and the bottom indicates the c-Jun-WT sequences, with the target site underlined.
